# Prevention of mother-to-child transmission of the Human Immunodeficiency Virus: investigating the uptake and utilization of maternal and child health services in Tiko health district, Cameroon

**DOI:** 10.11604/pamj.2015.20.20.5137

**Published:** 2015-01-07

**Authors:** George Awungafac, Patrick Achiangia Njukeng, Juliana Ajoache Ndasi, Lawrence Tanyi Mbuagbaw

**Affiliations:** 1 Global Health Systems Solutions (GHSS), SONARA Road, Limbe, Cameroon; 2 University of Dschang, Dschang, Cameroon; 3 Faculty of Health Sciences, University of Buea, Buea, Cameroon

**Keywords:** Antenatal care, PMTCT, MCH, Cameroon

## Abstract

**Introduction:**

Despite evidence that interventions to prevent mother-to-child transmission (PMTCT) of HIV are effective in ensuring a healthy child and keeping mothers alive, there are many challenges to achieving successful interventions in Cameroon. The study was conducted to investigate factors that affect access to and utilization of maternal and child health (MCH) and PMTCT services among women in Tiko health district in Cameroon.

**Methods:**

We conducted a cross-sectional, descriptive study on women of reproductive age who had experienced a pregnancy using a self-administered, structured questionnaire, in health facilities offering PMTCT services and in communities within the district.

**Results:**

Four hundred and thirteen women were interviewed. The majority, 98.4%, of them attended antenatal care (ANC) during their most recent pregnancy. Of these women, 87.4% of them made at least four ANC visits. HIV testing during the first visit among the ANC attendees was 85.5%. Approximately, 92.1% of women who tested for HIV received their results on the same day. All participants reported to have given birth in a health facility during their most recent pregnancy. No education (Odds Ratio [OR] 0.11; 95% CI 0.01-0.83) and acquisition of primary education (OR 0.25; 95% CI 0.06-0.88) was associated with better male partner involvement in PMTCT.

**Conclusion:**

The uptake of MCH/PMTCT services was high in this study. Further exploration of these levels is warranted so that this model of care and engagement can be replicated in other parts of the country where uptake is low.

## Introduction

Globally, approximately 289,000 maternal deaths occurred in 2013 [1]. Sub-Saharan Africa accounts for about 98% of maternal deaths in Africa [2]. In 2010, 64% of the 7.6 million deaths among children less than 5 years were attributable to infectious disease etiologies. [3]. The human immunodeficiency virus (HIV) and the acquired immune deficiency syndrome (AIDS) were associated with 1.8 million deaths including 250,000 among children less than 15 years in 2010 [4]. In Cameroon, AIDS-related diseases were associated with 980 maternal deaths in 2010 meanwhile the same year, 5% of under-5 mortality was linked to HIV/AIDS [5]. Maternal and child health outcomes have been shown to be linked to the quality of antenatal care (ANC), delivery services and post-natal care [2, 6–8]. Prevention of mother-to child HIV transmission (PMTCT) strategies introduced in 2001, [9] have become integrated into all maternal and child health services. Antenatal care (ANC) is a major entry point for PMTCT programs especially in countries with a high prevalence of HIV. The World Health Organization (WHO) recommends four ANC visits during pregnancy [10], which allows for early diagnosis, prevention or treatment of conditions which may jeopardize the health of the mother or her unborn baby.

High maternal, neonatal and child mortality rates are associated with inadequate and poor-quality maternal health care, reflected in the “three delays”; deciding to seek care, reaching the health care facility in time and receiving adequate treatment [11]. In the absence of intervention 50% of children born to HIV positive mothers will die before reaching their second birthday [12, 13]. The challenges facing the PMTCT programs in sub-Saharan Africa are numerous including large proportions of home deliveries, fear of the cultural implications of a positive HIV test result, such as the lack of male partner support and even violence [14–17]. In the context of an HIV epidemic, the WHO developed a four-prong approach to optimize the effectiveness of PMTCT interventions aimed at improving maternal and child health, which include primary prevention of HIV among women of reproductive age, prevention of unintended pregnancies among HIV infected women, PMTCT interventions and provision of care, treatment and support for children, mothers and affected families.

In Cameroon there has been a slow increase in the rate of antenatal care attendance form 5.1% in 2004 to 38.1% in 2010 [18]. This trend in ANC use in Cameroon has been shown to be associated with low uptake of other essential maternal and child health (MCH) services, such as skilled attendance at birth [15] and PMTCT strategies. According to records from the Cameroon health ministry (MOH), there has been an improvement in the percentage of deliveries conducted by skilled birth attendants from 61% in 2004 to 64% in 2011 [18]. In 2004, 59% of pregnant women delivered in a health facility increasing to 61% in 2011. The potential for mother-to-child transmission (MTCT) could be high with traditional birth attendance. Results from the Early Infant Diagnosis (EID) programs indicate an HIV prevalence of 8.8% among infants born to HIV positive mothers [19]. Acknowledging a high HIV prevalence (8.0%) among pregnant women in Cameroon [20], and the potential risks of MTCT, an increase in MCH service use in health facilities with PMTCT services will improve maternal and child health. The government of Cameroon and foreign partners including the Unites States, through the President's Emergency Plan for AIDS Relief (PEPFAR) has intensified efforts to fight the HIV epidemic in Cameroon in the ongoing PMTCT programs. In a setting like Cameroon with ethnic diversities, many factors can affect a woman's ability to seek MCH and PMTCT services and hence adopt positive behaviors. These may include marital status, socio-economic status, age, parity, history of delivery complications, religion and cultural factors [21, 22]. Limited data exist on these factors in Cameroon.

Identifying factors influencing uptake of MCH/PMTCT will inform policies and programs to help improve maternal and child health outcomes. The cascade for diminishing services used at PMTCT/MCH services demonstrates that Cameroon is and may continue to face key public health challenges for MCH/PMTCT. Considering national and global interest in the millennium development goals 4 (reduce child mortality), 5 (improve maternal health) and6 (combat HIV/AIDS, malaria, tuberculosis and other diseases) [23], understanding these factors is crucial. Also knowledge of barriers will ensure that women and children are retained in prevention, treatment and care services in Cameroon. The objectives of this study were to investigate the use of MCH/PMTCT services in health district setting (Tiko) in Cameroon.

## Methods

### Study ethics

The study was commenced after ethical approval by the University of Buea, Faculty of Health Sciences Institutional Review Board (Ref: 2012\0047\UB\FHS\IRB). All eligible participants gave their approval to participate by signing the consent form before the interview

### Design and study population

This was a cross-sectional and descriptive study, conducted over a period of six months between January and June 2012. It involved women of reproductive age selected from two settings; within the communities and at the health facilities offering ANC and PMTCT services in the Tiko Health District (THD). The advantage of recruiting women from both settings balances the possible problem of retention in ANC among women who may start but fail to continue in the cascade of services. Also, mothers who have never attended ANC and other MCH services can only be identified in the communities, where it is more complex to understand certain health seeking behaviors like use of traditional MCH health services.

### Study setting

The Tiko health district (THD) is situated in Fako division in the South West Region of Cameroon. Fako division is one of the 6 administrative divisions of the South West and has 4 health districts among which isTiko. The THD is made up of eight health areas each having a health centre. Tiko is a cosmopolitan geographic setting with both rural and semi-urban segments. It has a surface area of 484 square kilometers (sq.km), a population density of 241 inhabitants per sq.km and a population growth of 2.9%.

### Inclusion criteria

All pregnant women and mothers of reproductive ages, 15-49years.

### Exclusion criteria

The following women were not included in the study: Absence of the legal representatives for women aged 15-21 years; Participants less than 15 years of age; Participants who could not provide consent.

### Sample size

To estimate the minimum sample required for the study, we assumed p (38.1%) [18] to be the uptake at first ANC in Cameroon, a minimum allowed sampling error (d) of 5% and z at 95% confidence interval (1.96). Substituting into z^2^×p(1-p)/d^2^ [24] gave a minimum sample of 363 participants. To improve on the power of study findings and also minimize effect of nonresponse by some of the women, the sample size was extended by 20%, to 436 participants.

### Sampling techniques

In the communities a two-staged cluster sampling method was used to identify participants. In stage one, the sampling frame consisted a list of all the quarters within the district. The quarter is the smaller administrative unit under the leadership of the quarter head. After identifying the quarters, households were randomly selected from where the participants could be identified. The women who met the inclusion criteria were administered face-to-face interviews, which explored socio-demographic factors (such as the age, educational level, marital status, occupation, religion and the number of children), their utilization of services for antenatal care, delivery and post-partum care of their infants. Their knowledge of PMTCT, and cultural practices related to MTCT was entered into a structured questionnaire. To assess the attitude of women and their partners in activities towards PMTCT, women were asked if partners support HIV testing, accompany them to ANC, if they know of their partners’ HIV status and whether they discuss testing with their partners. Women received information about the study during ANC visits and infant welfare clinics and during prior visits to the community before commencement of data collection. The questionnaire was administered at the following primary healthcare facilities: Mutengene Sub-Divisional Hospital, the Tiko Central Clinic (TCC), Cottage Hospital, and the Holtfort Integrated Health Centre.

### Data Analysis

Data were entered and analyzed with Epi Info statistical software version 3.5 [25]. The independent variables included; socio-demographic elements (age, marital status, occupation, educational level, and number of children). The outcome variables measured were; knowledge of ANC/PMTCT, attendance at ANC, male partner involvement, acceptance of HIV testing, collecting HIV test results the same day, and disclosure of HIV status to partner. Relationships between the variables were established using the Chi-Square test at p

## Results

### Socio-demographic features of participants

Four hundred and thirty-six women were contacted. Of these, 413 signed the consent form and participated in the interview, giving a response rate of 94.7%. The minimum age of the participants was 17 years and the maximum age was 44 years (range = 27 years) with a mean age of 27.8 years. Table 1 displays detailed socio-demographic features of the respondents.

**Table 1:** Socio-demographic characteristics of 413 respondents

Characteristic, n = 413	Frequency N (%)	Mean ±SD
**Age (years)**		
17-24	142 (34.4%)	23.69 ± 1.35
25-34	225 (54.4%)	28.17 ± 3.59
35-44	43 (10.5%)	10.5 ± 3.97
**Education**		
None	25 (6.2%)	
Primary	248 (60.0%)	
Secondary	107 (25.9%)	
Post-secondary/university	33 (7.9%)	
**Occupation**		
Unemployed	128 (31.1%)	
Farming	55 (13.4%)	
Business	72 (17.4%)	
Hairdressing	33 (7.9%)	
Tailoring	70 (17.0%)	
Others	54 (13.1%)	
**Marital status**		
Married	278 (67.2%)	
Single	112 (27.2%)	
Co-habiting	23 (5.6%)	
**Number of children1.89 ± 1.44**		
1-3	295 (71.5%)	
More than 3	55 (13.4%)	
No child	63 (15.1%)	

### Use of Antenatal care, delivery and post-natal services

Overall, antenatal care attendance among the 413 women was high, and 406 women (98.4%) attended at least one ANC visit. Table 2 displays the use of antenatal care services in THD The main reason for not attending ANC was financial in 5 (66.7%). All the women, 413 (100%), reported to have delivered in a health facility in the THD during their most recent pregnancy. All the women, 413 (100%), including those that never attended ANC attended post natal visits for infant welfare activities.

**Table 2:** The use of antenatal care services among women in Tiko

Characteristic	Number (%)
**Timing of first ANC**	
First Trimester	59 (14.4)
Early Second Trimester (3-4months)	332 (80.3)
Third Trimester	22 (5.2)
**Number of ANC visits**	
4 visits	361 (87.4)
2 visits	26 (6.2)
1 visit	12 (0.3)
No visit	7 (1.7)

### Attitudes towards HIV testing

Nine (2.3%) of the 413 women had never done an HIV test before. The majority of them (355 or 85.9%) had done the test during their last pregnancy, while 8 (2.0%) tested before they were married and 124 (30%) had tested at other times. The women were asked if they were aware of HIV testing during antenatal clinics. Fifty nine (14.4%) of them had never heard about HIV testing during an ANC visit. Those that were aware got the knowledge from family members (168 or 40.7%), health personnel (161 or 39.0%), radio or public program (21 or 5.2%) and from the church (3 or 0.7%). Among the women, 12 (3.0%) did not do an HIV test during the most recent pregnancy. Majority (392 or 94.8%) of them accepted to do an HIV test during their first ANC visit. Five (1.3%) of them tested at other ANC visits. Three hundred and eighty (92.1%) of those who tested collected the test result on the same day. Eleven (2.6%) did not collect their results and 21 (5.2%) of the women collected the results at some other time after testing. Among the women, 263 (63.6%) tested for HIV due to fear of an HIV infection. Some tested because they do not trust their partners (39 or 9.5%) and in order to prevent MTCT (12 or 3.0%). Sixty-four (64, 15.4%) women tested under the influence of the health workers.

### Knowledge of MTCT and prevention

Table 3 displays the responses of the women to key questions on PMTCT.

**Table 3:** Responses of participants to five questions to assess current knowledge of PMTCT

Current knowledge	Correct answer N (%)
1. Ever heard of PMTCT	311 (75.4)
2. HIV positive woman can transmit the virus to her fetus during pregnancy	195 (47.2)
3. HIV positive woman can transmit the virus to her child during labor	138 (33.4)
4. HIV positive woman can transmit the virus to her child during breastfeeding	203 (49.2)
5. ARVs given to an HIV infected pregnant woman can reduce the chances of transmitting HIV to the fetus	222 (53.8)

### Role of male partner in maternal and child health activities

Table 4 displays the perceived role played by male partners in MCH activities in Tiko. The table reveals that 69 (16.8%) of male partners are reported to have accompanied their female counterparts for HIV testing during ANC.

**Table 4:** Male partner involvement in maternal and child health activities in Tiko

Variable	n = 413 N (%)
Accompany wife to ANC	
Yes	69 (16.8)
No	344 (83.2)
Husband ever tested for HIV	
Yes	200 (48.5)
No	83 (20.0)
Don't know	130(31.5)
Discuss HIV testing with partner	
Yes	261 (63.3)
No	152 (36.7)
Partner supports HIV testing	
Yes	320 (77.5)
No	70 (16.9)
Don't know	23 (5.6)

### Health system-related factors

Of the 413 women, 71 (17.1%) were requested by the health workers to come with partners for HIV testing at antenatal clinic. Emphasis on HIV testing at ANC is perceived as “not strong” by the majority of the women (355 or 82.9%). Most of the clients (366 or 88.5%) trust the health facility staff. The cost of ANC services is perceived to be acceptable by most of the women (405 or 98.0%).

### Factors associated with the use of MCH services in Tiko Health District

Table 5 displays various factors associated with the use of MCH services in Tiko. The results show that low level of education (primary and below) was associated with improved male involvement in MCH/PMTCT activities (no education: OR 0.11, 95% CI 0.01-0.83; Primary: OR 0.25, 95% CI 0.06-0.88). Partners of women who discuss HIV testing freely were more likely to accompany them to ANC for HIV testing and mutual disclosure of results (OR 4.06, 95% CI 1.76-9.33).

**Table 5:** Association between factors that influence the use of MCH services in Tiko

Variable	n	Odds Ratios OR (95% CI)	p-value
**Socio-demographic factors and male participation in MCH activities**			
**Level of education**			
No education	25	0.11 (0.01-0.83)	0.039
Primary	248	0.25 (0.06-0.88)	0.034
Secondary	107	0.33 (0.09-1.17)	0.092
Post-secondary	33	0.42 (0.03-5.16)	0.501
**Marital status**			
Married	278	2.97 (0.35-25.10)	0.323
single	112	2.58 (0.29-22.78)	0.390
**Occupation**			
Farming	55	2.05 (0.58-7.29)	0.271
Hairdressing	33	0.63 (0.07-5.89)	0.682
Teaching		5.43 (0.80-36.83)	0.080
Tailoring	70	3.92 (1.17-13.13)	0.023
Unemployed	128	2.35 (0.75-7.34)	0.142
**Discuss HIV testing with partner, and knowledge of PMTCT Partner came to facility for HIV testing during ANC**			
Yes	69	4.06 (1.76-9.33)	0.001
**ARVs given to HIV positive mother can reduce MTCT**			
Yes	222	1.37 (0.80-2.34)	0.251
No	191	0.49 (0.09-2.49)	0.392
**HIV positive pregnant women can transmit to unborn child during pregnancy**			
Yes	195	1.06 (0.59-1.90)	0.834
No	218	1.02 (0.41-2.56)	0.971
**HIV positive pregnant women can transmit to unborn child during labor**			
Yes	138	1.44 (0.79-2.62)	0.227
No	275	0.79 (0.35-1.78)	0.562
**HIV positive pregnant women can transmit to unborn child during breastfeeding**			
Yes	203	1.60 (0.91-2.83)	0.106
No	210	0.87 (0.29-2.56)	0.812

## Discussion

The uptake of services for MCH was high in this study. This is reflected in the high ANC attendance, good uptake of HIV testing at ANC, and all deliveries being conducted at the health facility and infant follow up visits. Male partner involvement in MCH/PMCT was high in this study. As many countries continue to roll out programs aimed at increasing access to MCH/PMTCT services, it is crucial to investigate potential barriers to implementation of these essential services. This study identified potential barriers and facilitators of implementation of ANC/MCH/PMTCT program in Tiko health district, Cameroon.

Our results showed that the level of utilization of healthcare facilities for ANC/PMTCT/MCH services among women in Tiko is high. This is indeed more than utilization of maternal health services in many other Sub-Saharan countries. For example we found in this study that 98.4% of women had one ANC visit and 87.4% have the recommended four ANC visits during ANC services during their most recent pregnancy. Some comparative values are 60.3% for Nigerian mothers [2], 91.9% for Ghana, 72.8% for Burkina Faso and 88.0% for Benin [26]. The finding that skilled delivery services is higher (100%) than use of ANC services is however not consistent with the results of other studies conducted. For example the study of Okafor and colleagues [27] on use of maternal health services in Nigeria and Francis and Michel [28] in a study in Uganda found that 39% of the women delivered at home and up to about 70% of rural women in Uganda deliver at home [29]. The ANC and high skilled health facility delivery in Tiko district may be due to the US-Government President Emergency Plan for Aids Relief (PEPFAR) PMTCT program presently being implemented by the Baptist Mission Health Board. This program has provided easy accessibility to PMTCT and antiretroviral therapy (ART) programs to most of the healthcare facilities in the South West Region.

The readiness to accept an HIV test at ANC was high in this study. High rates of access to HIV testing and uptake of testing at ANC have been reported since the initiation of the PMTCT program in Cameroon and stood at 79% in 2010 [19]. The finding of 85.7% HIV testing rate at ANC is consistent with national interest in achieving the Millennium Development Goal (MDG) 6 in 2015. Majority of the women interviewed preferred to collect their HIV test results on the same day, some collected the results later meanwhile others never collected their results. It has been shown that same day results can be provided in counseling without compromising the quality of counseling [27] and is a strong impetus for client compliance and retention in ANC services. Since some of the women tested under the influence of the health workers, it is likely that those who do not receive their results are those who are coerced to accept testing or who have been told to come back for results. One major challenge identified in this study is the poor knowledge of MTCT. This knowledge may be attributable to poor socio-demographic parameters like age, educational level, occupation and poor use of the media which is currently a medium to deliver messages on HIV and the ongoing effort of PMTCT. Very few women in this study had ever heard of PMTCT/HIV talks on the radio. Knowledge of PMTCT has been shown to correlate with increased use of the media [27].

It has always been assumed that where couples undergo HIV testing and mutual disclosure of results there is increased acceptability of results, improved adherence to ARVs and a significant change in health behavior [30, 31]. For example in Uganda, counselor-assisted disclosure for couples in their home or at a facility is being piloted. In Kenya, partner disclosure by women with HIV has been associated with a 4-fold increase in reported condom use to nearly 70%. Partner notification for sexually transmitted infections has been implemented in some African countries [32]. This study illustrates that participation of male partners in the ANC/MCH/PMTCT in Tiko is 16.8%. This is consistent with a study in North West region of Cameroon, which showed that male participation in PMTCT activities had never exceeded 17% (20). However, compared with other countries in Africa [31, 33], this double-digit figure in Cameroon is high. While male involvement is a welcomed development with potential to improve MCH indices, the relatively high figures in this study merits closer reviews to better understand the true trend and associated factors. A policy was adopted by the ministry of health in Cameroon (MOH) whereby partners of all women attending ANC are invited by the health personnel to do HIV testing for mutual disclosure of results among couples. This is not the case in majority of the respondents as only about 17% of the women were asked to bring partners for counseling on PMTCT. Cost of ANC services does not appear to be a major barrier from this study as many of the women report that services are offered at acceptable cost.

### Limitations of the study

This study does not explore healthcare providers’ perspectives on factors that affect uptake of MCH services in the district, which could have expose a complete picture on barriers to uptake of services both from the clients’ and services providers’ sides. The clients in this study may also confer information bias by over reporting their observations and thoughts.

## Conclusion

The uptake of ANC/PMTCT services in Tiko is relatively high and meets stakeholders’ recommendations. Low level of education appears to be a predictor of male involvement in MCH services in THD. Further exploration of the high levels in this study is warranted to understand associated factors, which could guide engagement of this model of care to other parts of Cameroon with low uptake.
